# Deficits in Long-Term Vaccine Immunity Among Childhood Cancer Survivors Despite Revaccination Programs

**DOI:** 10.3390/vaccines13060617

**Published:** 2025-06-06

**Authors:** Alexander Zadruzny, Eva Tiselius, Tiia Lepp, Teodora Aktas, Teghesti Tecleab, Samuel Hellman, Maja Jahnmatz, Anna Nilsson

**Affiliations:** 1 Division of Pediatric Cancer, Department of Women’s and Children’s Health, Karolinska Institutet, 171 77 Stockholm, Sweden; 2 Public Health Agency of Sweden, Department of Public Health Analysis and Data Management, 171 65 Solna, Sweden; tiia.lepp@folkhalsomyndigheten.se (T.L.); samuel.hellman@folkhalsomyndigheten.se (S.H.); 3 Public Health Agency of Sweden, Department of Microbiology, 171 65 Solna, Sweden; 4 Pediatric Oncology, ME1, Astrid Lindgren’s Children Hospital at Karolinska University Hospital Solna, 171 64 Solna, Sweden

**Keywords:** childhood cancer survivors, revaccination, tetanus, rubella, long-term immunity

## Abstract

Background: Childhood cancer survivors (CCSs) often experience impaired humoral immunity because of cancer treatments that increase their susceptibility to vaccine-preventable diseases. This study aimed to assess the seroprevalence of tetanus and rubella antibodies in CCSs compared to healthy, age-matched controls. Additionally, we explored the impact of cancer treatments on vaccine-induced immunity, examined the extent of revaccination after treatment completion, and evaluated the effectiveness of revaccination on seroprevalence. Methods: This retrospective study included 180 CCSs previously treated at Astrid Lindgren Children’s Hospital, Stockholm, between March 2019 and January 2023. Patient data were retrieved from electronic medical records. Seroprevalence data for rubella and tetanus antibodies in the 15–19-year age group were also obtained from a national seroprevalence study conducted by the Public Health Agency of Sweden. Results: CCSs exhibited significantly lower seroprevalence for both tetanus (77.7% vs. 92.7%) and rubella (79.1% vs. 97.5%) compared to age-matched controls. Revaccination with DTP-containing vaccines was more frequently administered than with the MMR vaccine. Tetanus and rubella seroprevalence were the lowest in children who had received intense chemotherapy. Among those who were revaccinated with the DTP vaccine after intensive treatment, 81 out of 98 (82.6%) had tetanus IgG levels above the threshold, compared to 24 out of 48 (50%) unvaccinated CCSs. In contrast, among those revaccinated with MMR, 57 out of 73 (78.1%) had positive rubella IgG, compared to 53 out of 73 (72.6%) unvaccinated CCSs with rubella IgG levels above the cut-off. Conclusions: Our findings highlight that vaccines are underutilized in CCSs with a notable gap in immunity, particularly among those who have undergone intensive treatments. Unexpectedly, MMR revaccination did not significantly affect rubella immunity. Given the increasing number of CCSs, it is essential to better understand how to effectively restore vaccine immunity in this population.

## 1. Introduction

Advances in cancer treatment and advances in supportive care have together resulted in an overall survival in childhood cancer, exceeding 80% in high-income countries [1]. Improvements in treatment include more effective and higher doses of existing drugs, multimodal treatment protocols, but also the introduction of novel targeted therapies and, more recently, immunotherapy [2]. With time, it has become evident that all systemic therapies against childhood cancers affect the immune system in some way. In addition, radiation or splenectomy may also impair immune function. Though late toxicity after childhood cancer has gained interest and resulted in a still-expanding research field, the long-term function of the immune system in childhood cancer survivors (CCSs) is still not fully characterized [3].

In addition to severe immune deficiencies during active treatment, several long-term defects in the humoral and cellular immunity of children treated for cancer have been identified, including persistent depletion of lymphocyte subsets and loss of antibodies (Abs) against vaccine-preventable diseases (reviewed in [4,5]). Although most published studies focus on children treated for leukemia, a decrease in seroprevalence for vaccine Abs has also been reported for children with solid tumors. Thus, due to the potential risk of contracting a vaccine-preventable disease among CCSs, many working groups and organizations recommend revaccination after completed cancer therapy [6,7]. In the evidence-based guidelines from the Infectious Disease Society of America (IDSA), the general recommendations are to start revaccination three months after chemotherapy, except for patients who received anti-B-cell Ab treatment, where the recommendation is to postpone revaccination until 6 months after therapy [8].

The Swedish national immunization program (NIP) offers vaccinations free of charge to all children residing in Sweden, with vaccinations carried out in nurse-led well-baby clinics and school health services. The vaccine containing diphtheria, tetanus, and pertussis antigens (DTP-containing vaccine) is first scheduled at 3, 5, and 12 months of age followed by booster doses (at 5 and at 14–15 years of age), and the measles–mumps–rubella vaccine (MMR) is given at 18 months of age, with a second dose at 7–8 years of age [9]. Historically, Sweden has had a high and stable vaccination coverage for DTP-containing and MMR vaccines through the NIP [10].

After completed childhood cancer treatment, the general national recommendation for follow-up after childhood cancer in Sweden is to revaccinate children post-treatment without prior testing [11], with varying timepoints and numbers of doses depending on the treatment the child has received (Appendix A). Administration of inactivated vaccines is recommended three months after completion of therapy while live attenuated vaccines are provided after six months post-treatment. In patients who have received what is considered highly immunosuppressive therapy, vaccination is postponed to 6 and 12 months, for inactivated and live vaccines, respectively, to allow for further immune recovery (Appendix A). Following revaccination, the child is then recommended to follow the NIP at the age-appropriate level [9]. Finally, children who were not fully vaccinated prior to their cancer diagnosis are recommended to start a full vaccination post-treatment.

Despite ongoing revaccination in the routine care of CCSs, there are few reports in the literature on whether the existing vaccination guidelines are successful in restoring their long-term serological memory to vaccine antigens. The primary aim of this study was to compare the seroprevalence of rubella and tetanus Abs in CCSs aged 16–19 years compared to age-matched controls at a single center in Sweden.

## 2. Materials and Methods

### 2.1. Study Cohort

This is a retrospective study of vaccine immunity in young adults born in the year 2000 or later and previously treated for cancer at the Pediatric Oncology Center at Astrid Lindgren Children’s Hospital, where all children (0–18 years of age) with cancer in the Stockholm region are diagnosed, treated, and followed.

According to Swedish guidelines, all patients are summoned for a final visit between the age of 16 and 18 years before referral to adult care. As part of the clinical routine, serology for tetanus and rubella is performed before the visit. In total, 220 former patients were identified to have attended the final visit to the outpatient clinic between 1 April 2019 and 31 January 2023. This study was approved by the Swedish Ethical Review Authority (2024-00157-01).

Within the scope of a seroprevalence study conducted by the Public Health Agency of Sweden, anonymous residual blood samples were collected from clinical chemistry laboratories in different Swedish regions. For the current study, rubella and tetanus seroprevalence for the age group 15–19 years were included. Ethical permission and use of informed consent for the control samples was not required, as judged by the regional Swedish Ethical Review Authority in Stockholm, as samples could not be traced back to the individual (the Swedish Ethical Review Act 2003:460).

### 2.2. Sample and Data Collection

Two to four weeks before the final visit, blood samples are routinely sent to the Department of Clinical Microbiology, Karolinska University Hospital, Stockholm, for serological analysis. Within the scope of this study, patient data were collected from the electronical medical chart and the following data were retrieved: (1) current Ab serostatus for tetanus and rubella, (2) cancer diagnosis, age at first diagnosis, and number of relapses, (3) modality of treatment (surgery, chemotherapy, radiation, or a combination of the three), and (4) whether revaccination had been administered after cancer treatment or not. The intensity of past cancer treatment and subsequent degree of immunosuppression was categorized into mild, moderate, and intense, as previously published [12].

### 2.3. Serological Assays

In CCS samples, serology for tetanus was performed using the Virion/Serion GmbHs kit SERION ELISA classic Tetanus IgG kit (Institut Virion\Serion GmbH, Wurzburg, Germany) according to the manufacturer’s instruction and further analyzed on Freedom EVOlyzer (Tecan Trading AG, Männerdorf, Switzerland). Serology for rubella was performed using X LIAISON^®^ Rubella IgG II (CLIA, Saluggia, Italy) and analyzed on a LIAISON^®^ XL analyzer (Diason, Saluggia, Italy). Seroprevalence was reported for individuals with an Ab level above positive level (≥10 IU/mL for rubella, ≥0.3 IU/mL for tetanus).

For the control samples, the presence of rubella virus-specific IgG Abs was analyzed using Euroimmun^®^ anti-rubella virus ELISA IgG according to manufacturer’s instruction [13]. For the detection of tetanus-specific IgG Abs, an inhouse multiplex assay, based on a previously published method [14], was used. Seroprevalence was determined as the weighted average of individuals with an Ab level above cut-off (≥11 IU/mL for rubella, ≥0.1 IU/mL for tetanus).

### 2.4. Statistical Analysis

Data were analyzed based on the primary cancer diagnosis, age at first diagnosis, and intensity of treatment. Data on age at diagnosis were categorized into three age groups: preschool children (0–5 years old), school children (6–12 years old), and teenagers (13–18 years old). Continuous data were analyzed with a Kruskall–Wallis test and adjusted for multiple testing according to Dunn’s multiple comparison test. Categorical variables were calculated with Fisher’s Exact Test. A *p*-value < 0.05 was considered significant. Statistical analyses were performed using GraphPad v 10.4.1 CA or SPSS Statistics, v 23 NY. Statistical analysis was not deemed appropriate for comparisons between CCSs and healthy age-matched controls due to the use of two different serological methods.

## 3. Results

### 3.1. Characteristics of the Study Population

Out of the 220 identified CCSs, 40 patients were excluded due to being lost to follow-up (*n* = 21), or having missing data on serostatus for both tetanus and rubella (*n* = 19), resulting in 180 patients included in the study population.

Table 1 shows the clinical characteristics of the CCS group; 106 children (59%) were diagnosed with leukemia/lymphoma including Hodgkin’s lymphoma, 51 children (28%) with a solid tumor, and 23 children (13%) with a CNS tumor. There were no differences in sex distribution between the diagnostic subgroups although there were slightly more boys (56.1%) than girls (43.9%) included. Furthermore, the age distribution at diagnosis between the different subgroups did not significantly differ (*p* = 0.057) though CNS tumors were more common in preschool children compared to both solid tumors and leukemia/lymphoma.

As expected, the estimated treatment intensity and subsequent immunosuppression differed significantly among the diagnostic subgroups (*p* < 0.001). The proportion of children exposed to intensive immunosuppression during their primary cancer treatment amounted to 48% for CNS tumors, 66% for solid tumors, and 95% for leukemia/lymphoma. A small number of children suffered from relapse, with no significant differences between the diagnostic subgroups (*p* = 0.496). Revaccination after completed therapy had been administered with a DTP-containing vaccine to a higher degree than MMR for all diagnostic groups, ranging from 39 to 68% of those vaccinated, compared to CCSs vaccinated with the MMR vaccine, ranging from 22 to 53%. Diagnosis had a significant impact on revaccination rates (for DTP *p* = 0.009 and MMR *p* = 0.011, respectively), where children with a previous diagnosis of leukemia/lymphoma were revaccinated to a larger extent.

### 3.2. Seroprevalence of Tetanus IgG and Rubella IgG

**Figure 1:**
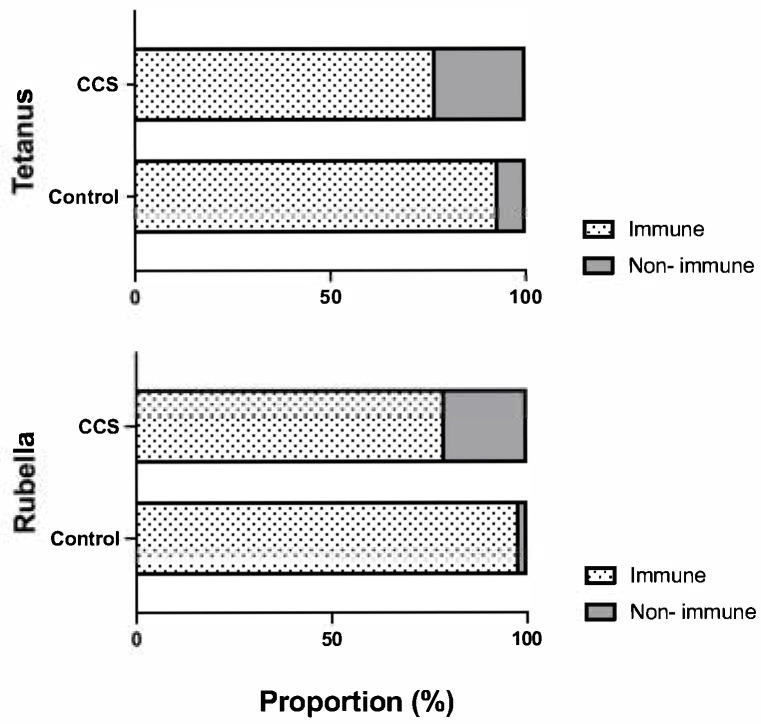
Seroprevalence of tetanus (**upper** panel) and rubella (**lower** panel) antibodies in childhood cancer survivors (CCSs) compared to age-matched controls from the general population. Immune was defined as antibody levels above the predefined cut-off for the respective assay.

The prevalence of IgG titers against tetanus and rubella was first compared between CCSs and age-matched controls from the general population. The CCSs showed lower seroprevalence for both tetanus (77.7% vs. 92.7%) and rubella (79.1% vs. 97.5%) at the final key visit compared to controls (Figure 1). Though patients with leukemia/lymphoma presented the lowest results, with 72.8% for tetanus and 76.5% for rubella, there was no statistically significant difference in the distribution of Ab prevalence when comparing between the diagnostic subgroups.

### 3.3. Status of Revaccination and Protective Antibodies Depending on Degree of Immunosuppression

Since the Swedish national guidelines for revaccination of CCSs recommend different schedules of revaccination depending on the treatment intensity (Appendix A), we also analyzed the outcome of revaccination based on treatment intensity (Table 2). Following completed cancer therapy, revaccination of the most intensely treated patients with a DTP vaccine was seen in 98 of 146 (67.1%) of CCSs, and with an MMR vaccine in 73 of 146 (50.0%) of CCSs. Similarly, the distribution of revaccination among moderately exposed patients showed a higher proportion of CCSs revaccinated with DTP compared to the MMR vaccine [9 of 27 (33.3%) and 5 of 27 (18.5%), respectively]. Notably, the mildly exposed patients stood out, as only a few patients had been revaccinated (one of seven CCSs with the DTP vaccine and two of seven with the MMR vaccine).

Of the children who received DTP revaccination after exposure to intense treatment, 81 out of 98 (82.6%) had tetanus IgG levels above cut-off in comparison with 24 out of 48 (50%) of the unvaccinated. In DTP revaccinated children previously exposed to mild (*n* = 1) and moderate (*n* = 9) treatment, all individuals had tetanus IgG levels above cut-off. Fifty-seven out of seventy-three (78.1%) MMR-revaccinated CCSs exposed to intense treatment had a positive rubella IgG while 53 out of 73 (72.6%) unvaccinated CCSs had rubella IgG levels above cut-off. For the mild and moderate group combined, the number of individuals with rubella IgG levels above cut-off in the revaccinated CCSs were five of seven individuals. In the unvaccinated mild and moderate group, 21 out of 27 had rubella IgG levels above cut-off (Table 2).

### 3.4. Association of Antibody Prevalence with Different Background Variables

Next, we examined whether factors such as age at diagnosis, cancer subgroup, treatment intensity, or revaccination influenced the prevalence of tetanus and rubella IgG in CCSs, both with and without Ab levels above cut-off (Table 3). Gender did not influence antibody prevalence.

Tetanus IgG above cut-off was associated with the intensity of previous treatments (*p* = 0.023), where a more intense treatment was associated with a lower proportion of CCSs with tetanus IgG. Moreover, revaccination increased the proportion of CCSs with tetanus IgG above cut-off [91/104 (87.5%)] compared to those who were not revaccinated, where only 26 out of 71 (36.6%) had tetanus IgG (*p* < 0.001).

Rubella IgG above cut-off was associated with age at diagnosis (*p* = 0.036), where children diagnosed at 6 to 12 years old were more likely to have rubella IgG. There was no correlation (*p* = 0.572) between revaccination after cessation of therapy and the presence of rubella IgG, as MMR revaccination did not significantly increase the seroprevalence of rubella IgG above cut-off in revaccinated individuals [62/76 (81.6%)] compared to those who missed revaccination [74/96 (77.1%)].

## 4. Discussion

In this retrospective cohort study, we show that CCSs aged 16–18 years old have a reduced seroprevalence of tetanus and rubella IgG compared to healthy age-matched individuals from the general population. Furthermore, we also found that previous cancer treatment intensity and age at diagnosis had an impact on seroprevalence. Of note, our results show that the current national guidelines on revaccination after childhood cancer are only partially followed. Additionally, regardless of revaccination rates, it is worrisome that revaccination does not fully restore or compensate for loss of humoral immunity during treatment in a group of young adults at risk for vaccine-preventable diseases [15].

It is well established that serological immunity to vaccination antigens is severely affected by modern chemotherapy protocols for a wide variety of childhood cancers [3]. Revaccination is therefore recommended in many countries to start three to six months after completion of therapy and has been in practice since 2004 in Sweden. Still, 20 years on, few studies have evaluated the long-term effect of these revaccination schedules. Previous studies focusing on children after completed therapy for acute lymphoblastic leukemia (ALL) have indicated that revaccination is needed but that it does not always induce stable Ab levels [16,17,18]. In a more recent paper, measles immunity was studied in CCSs who had been treated for childhood cancer several years previously [19]. Their results showed that CCSs with a history of two doses of MMR vaccine prior to cancer treatment and those with one dose prior and one after completion of therapy had low seroprevalence of measles IgG post-treatment. Interestingly, this contrasted with CCSs who had received two doses of MMR vaccine after completed therapy, where all individuals had Abs to measles upon testing. In our study, we observed a clear difference in seroprevalence between CCSs and age-matched 16 to 18-year-old controls and therefore went on to examine to what extent CCSs had been revaccinated or not.

Our cohort was dominated by CCSs previously treated for leukemia or lymphoma and who therefore had experienced intense chemotherapy at a young age. Despite an overall lack of adherence to the national guidelines of revaccination for all CCSs, independently of age and initial diagnosis, more than 50% of CCSs with leukemia or lymphoma had received revaccinations. In contrast, in CCSs treated for a CNS or solid tumor, less than half had been revaccinated according to guidelines. Previous studies have indicated a similar poor compliance by health care institutions to vaccination programs in this vulnerable patient group [20,21], with physician recommendation being identified as one of the most important factors for high vaccine up-take in CCSs [22,23]. Whether children had been offered revaccination and declined or simply not offered at all was not possible to elucidate in this study; neither do we know whether they had followed the NIP at the age-appropriate level post cancer treatment.

To further understand whether revaccination can restore long-term immunity, we also examined revaccination in relation to treatment intensity. For tetanus, a high proportion of revaccinated CCSs with previous intense treatment had Ab levels above the cut-off while only half of nonvaccinated CCSs had levels above cut-off. In the mild-to-moderate group, all revaccinated children had tetanus IgG above cut-off and most nonvaccinated CCSs were also considered above cut-off. It should be noted that intensely treated children most often also have a lengthy treatment period, as exemplified by ALL treatment for >2 years. Due to the limited cohort size, we did not stratify for long or short treatment periods. Interestingly, MMR revaccination in the intensely treated patients did not increase the proportion of CCSs with rubella IgG above cut-off compared to the non-revaccinated CCSs. This was an unexpected finding, as the MMR vaccine is reported to confer 95–98% seroconversion for rubella after the first and second dose in healthy individuals, with a very low subsequent waning rate [24]. Additionally, recent data on revaccination of children with ALL also showed an increase in seroprevalence of rubella IgG from 63 to 96% one month after a booster dose [25]. Within our study, mild and moderate-treated CCSs showed a higher proportion of individuals above cut-off for rubella, similar to tetanus. From these data, it is clear that treatment intensity plays a major role in persistent vaccine Ab levels after childhood cancer as previously suggested [26,27].

Whether the low seroprevalence of rubella IgG several years after revaccination in our cohort is due to a rapid waning or merely a failure to establish long-term plasma cells in the bone marrow remains unknown. Additionally, one can also speculate whether revaccination was provided at a timepoint when the immune system had recovered from previous treatment or not. Unfortunately, data describing immune reconstitution after standard chemotherapy in childhood cancer patients are scarce and sometimes conflicting (reviewed in [4]). Some publications have described normalization of lymphocyte counts already at three to six months [27], while other studies have shown a slower recovery of memory B- and T-cells [27,28]. It is, however, likely that immune reconstitution is a more complex process in children with hematological malignancy who received intense chemotherapy.

An additional important factor to take into consideration when evaluating outcomes of revaccination post-treatment is age at cancer diagnosis. In this cohort, in contrast to treatment intensity and revaccination, age at diagnosis did not seem to affect tetanus IgG serostatus at 16–18 year in CCSs. In most children within our cohort, primary immunization with three doses of DTP at 3, 5, and 12 months would have been given before the cancer diagnosis, with some children also receiving the additional dose at 5 years [9]. Still, with regard to rubella, age did seem to have an effect in children who were diagnosed with cancer at 6 to 12 years old, where a positive rubella serostatus was more commonly found at follow-up. This could possibly be explained by many children in the group having most likely received two doses of MMR vaccines before chemotherapy, in contrast to the younger age group [9].

Our study has some limitations and, most importantly, we were unable to retrieve a detailed history of immunizations for our CCS cohort. On the other hand, data from the healthy-age matched controls were also collected without knowledge of vaccination status, which is why we believe that the observed differences in tetanus and rubella serostatus stand true. In addition, we were not able to control for CCSs who were lost to follow-up and never attended the final visit. Unfortunately, the fact that their serostatus was evaluated with different laboratory methods, though both well established, did not allow for a statistical comparison between CCSs and their controls.

## 5. Conclusions

Our findings reveal that vaccines are underutilized among CCSs. There was considerable variability in the rates of revaccination based on the type of malignancy and treatment intensity. Notably, vaccine immunity was most commonly lacking in children who had undergone more intensive treatments. Unexpectedly, MMR revaccination did not affect rubella immunity in our cohort. As the population of CCSs continues to grow, it is crucial to enhance our understanding of how to effectively restore vaccine immunity in this vulnerable group.

## Figures and Tables

**Table 1:** Characteristics of study participants categorized by cancer diagnosis.

	Solid Tumors	CNS Tumors	Leukemia/Lymphoma *	
	*n* = 51	*n* = 23	*n* = 106	
**Age at diagnosis (years)**	8 (0–17 )	5 (0–14)	7.5 (0–17)	*p* = 0.056
median (range)				
Preschool (0–5 yrs) *n* (%)	*22 (43)*	*12 (61)*	*83 (46)*	
School (6–12 yrs) *n* (%)	*17 (33)*	*8 (35)*	*54 (30)*	
Teenagers (13–18 yrs) *n* (%)	*12 (24)*	*1 (4)*	*43 (24)*	
**Follow-up time (years)**	9 (1–17.5)	13 (2–18)	10.5 (1–18.5)	*p* = 0.078
median (range)				
**Estimated treatment intensity ** ***n*** ** (%)**				*p* < 0.001
Mild	1 (2)	1 (4)	5 (5)	
Moderate	16 (32)	11 (48)	0 (0)	
Intensive	34 (66)	11 (48)	101 (95)	
**Relapse *n* (%)**	6 (12)	3 (13)	8 (7)	*p* = 0.496
**Revaccination *n* (%)**				
DTP	25/51 (49%)	9/23 (39%)	72/106 (68%)	*p* = 0.009
MMR	19/51 (37%)	5/23 (22%)	56/106 (53%)	*p* = 0.011

* Including Hodgkins’s lymphoma.

**Table 2:** Seroprevalence in childhood cancer survivors with or without DTP and MMR revaccination.

Intensity of Treatment	Mild	Moderate	Intense
	*n* = 7	*n* = 27	*n* = 146
**DTP ** ***n*** ** (%)**			
**Revaccination**	*n* = 1	*n* = 9	*n* = 98 *
Tetanus IgG > cut-off	1 (100.0)	9 (100.0)	81 (82.6)
Tetanus IgG < cut-off	0 (0.0)	0 (0.0)	13 (13.2)
No revaccination *	*n* = 6	*n* = 18 *	*n* = 48
Tetanus IgG > cut-off	5 (83.3)	16 (88.8)	24 (50.0)
Tetanus IgG < cut-off	1 (17.7)	1 (5.6)	24 (50.0)
**MMR ** ***n*** ** (%)**			
**Revaccination**	*n* = 2 **#**	*n* = 5 **#**	*n* = 73 **#**
Rubella IgG > cut-off	1 (50.0)	4 (80.0)	57 (78.1)
Rubella IgG < cut-off	0 (0.0)	0 (0.0)	14 (19.2)
**No revaccination**	*n* = 5	*n* = 22 **#**	*n* = 73 **#**
Rubella IgG > cut-off	5 (100.0)	16 (72.7)	53 (72.6)
Rubella IgG < cut-off	0 (0.0)	5 (22.7)	17 (23.2)

* Tetanus: missing serological data in 4 patients after revaccination and 1 without revaccination. # Rubella: missing serological data in 5 patients after revaccination and in 3 without revaccination.

**Table 3:** Factors associated with vaccine immunity for tetanus and rubella in childhood cancer survivors.

	Tetanus			Rubella		
	IgG < Cut-off (*n* = 39)	IgG > Cut-off (*n* = 136)	*p*-Value	IgG < Cut-off (*n* = 36)	IgG > Cut-off (*n* = 136)	*p*-Value
**Age at diagnosis, *n***			0.160			**0.036**
0–5 years	22	60		21	57	
6–12 years	7	46		5	48	
13–18 years	10	30		10	31	
**Diagnosis group, *n***			0.175			0.587
Solid tumors	7	42		8	41	
CNS tumors	4	19		4	17	
Leukemia/lymphoma	28	75		24	78	
**Treatment intensity, ** ***n***			**0.023**			0.667
Mild	1	6		0	6	
Moderate	1	25		5	20	
Intense	37	105		31	110	
**DTP revaccination ** *, *n*			**<0.001**			
Yes	13	91		n/A	n/A	
No	26	45		n/A	n/A	
**MMR revaccination #, ** ***n***						0.572
Yes	n/A	n/A		14	62	
No	n/A	n/A		22	74	

* Tetanus: missing serological data in 4 patients after revaccination and 1 without revaccination. # Rubella: missing serological data in 5 patients after revaccination and in 3 without revaccination.

## Data Availability

The data presented in this study are available on request from the corresponding author, to adhere to the Swedish ethical review act (2003:460).

## Abbreviations

The following abbreviations are used in this manuscript:

CCSs	childhood cancer survivors
Abs	antibodies
NIP	national immunization program
DTP	diphtheria–tetanus–pertussis
MMR	measles–mumps–rubella
IgG	immunoglobulin G
CNS	central nervous system
ALL	acute lymphoblastic leukemia

## Appendix A

Standard intensity High intensity
